# Supplementary material for: Evaluation of the psychometric properties of the Episodic Disability Questionnaire (EDQ) among women living with HIV in the United Kingdom: A self-reported repeated measure study
Source: PLoS One. 2026 May 4;21(5):e0336679. doi: 10.1371/journal.pone.0336679 (PMC13138649; doi:10.1371/journal.pone.0336679)
Supplement: S1 Table — (DOCX) [file pone.0336679.s001.docx]

**S1 Table. Participants’ additional demographic, health, and social characteristics**

| **Primary language** (n=103) | **Number (%)** |
| --- | --- |
| English | 88 (85%) |
| Other language including British Sign language | 15 (15%) |
| **English language proficiency** (n=103) | **Number (%)** |
| Very well | 85 (83%) |
| Well | 17 (17%) |
| Not well at all | 1 (1%) |
| **General self-rated health status** (n=101) | **Number (%)** |
| Excellent | 8 (8%) |
| Very good | 22 (22%) |
| Good | 33 (33%) |
| Fair | 32 (32%) |
| Poor | 6 (6%) |
| **General health status compared to 1 year ago** (n=101) | **Number (%)** |
| Much better now than one year ago | 13 (13%) |
| Somewhat better now than one year ago | 13 (13%) |
| About the same as one year ago | 50 (50%) |
| Somewhat worse now than one year ago | 20 (20%) |
| Much worse now than one year ago | 5 (5%) |
| **Common concurrent health conditions**  **(≥30% of sample)** | **Number (%)** |
| Chronic pain - Joint | 45 (45%) |
| Chronic pain - Soft tissue | 42 (42%) |
| High cholesterol | 41 (41%) |
| Mental health condition (depression, anxiety) | 37 (37%) |
| Trouble sleeping | 36 (36%) |
| High blood pressure | 36 (36%) |
| Problems with sexual pleasure | 34 (34%) |
| Migraines or headaches | 32 (32%) |
| **Substance use in past 30 days** (n=101) | **Number (%)** |
| Cigarette (tobacco, pipe, cigar) - I am a former smoker (have not smoked in the last 30 days) | 14 (14%) |
| Cigarette (tobacco, pipe, cigar) - I currently smoke (in the last 30 days) | 5 (5%) |
| Cigarette (tobacco, pipe, cigar) - I smoke occasionally (in the last 30 days) | 1 (1%) |
| Alcohol | 45 (45%) |
| Cannabis | 1 (1%) |
| Methamphetamines | 0 (0%) |
| Cocaine | 0 (0%) |
| Heroin | 1 (1%) |
| Non-prescribed opioids | 0 (0%) |
| Other substance use | 3 (3%) |
| **Have sufficient money to cover basic needs (food, clothes, heating, accommodation)** (n=103) | **Number (%)** |
| Most of the time | 37 (36%) |
| All of the time | 30 (29%) |
| Some of the time | 25 (24%) |
| No | 11 (11%) |
| **Relationship status** (n=103) | **Number (%)** |
| Single, never married or never registered a civil partnership | 35 (34%) |
| Married | 29 (28%) |
| Divorced | 14 (14%) |
| Widowed | 12 (12%) |
| Separated, but legally still married | 7 (7%) |
| In a registered civil partnership | 4 (4%) |
| Formally in a civil partnership which is now legally dissolved | 2 (2%) |
| **Care and support responsibilities for others** (n=103) | **Number (%)** |
| No | 70 (68%) |
| Yes, 1-19 hour a week | 20 (19%) |
| Yes, 20-49 hours a week | 11 (11%) |
| Yes, 50 or more hours a week | 2 (2%) |
