# Supplementary material for: Evaluation of the psychometric properties of the Episodic Disability Questionnaire (EDQ) among women living with HIV in the United Kingdom: A self-reported repeated measure study
Source: PLoS One. 2026 May 4;21(5):e0336679. doi: 10.1371/journal.pone.0336679 (PMC13138649; doi:10.1371/journal.pone.0336679)
Supplement: S2 Table — (DOCX) [file pone.0336679.s002.docx]

**S2 Table. EQ-5D-5L frequency (%) of scores per domain (n=103)**

| **Mobility** | **Number (%)** |
| --- | --- |
| I have no problems in walking about | 52 (50%) |
| I have slight problems in walking about | 15 (15%) |
| I have moderate problems in walking about | 24 (23%) |
| I have severe problems in walking about | 12 (12%) |
| I am, unable to walk about | 0 (0%) |
| **Self-care** | **Number (%)** |
| I have no problems washing or dressing myself | 71 (69%) |
| I have slight problems washing or dressing myself | 14 (14%) |
| I have moderate problems washing or dressing myself | 11 (11%) |
| I have severe problems washing or dressing myself | 7 (7%) |
| I am unable to wash or dress myself | 0 (0%) |
| **Usual activities (e.g. work, study, housework, family or leisure activities)** | **Number (%)** |
| I have no problems doing my usual activities | 51 (50%) |
| I have slight problems doing my usual activities | 25 (24%) |
| I have moderate problems doing my usual activities | 17 (17%) |
| I have severe problems doing my usual activities | 9 (9%) |
| I am unable to do my usual activities | 1 (1%) |
| **Pain / discomfort** | **Number (%)** |
| I have no pain or discomfort | 21 (20%) |
| I have slight pain or discomfort | 34 (33%) |
| I have moderate pain or discomfort | 32 (31%) |
| I have severe pain or discomfort | 14 (14%) |
| I have extreme pain or discomfort | 2 (2%) |
| **Anxiety / depression** | **Number (%)** |
| I am not anxious or depressed  I am slightly anxious or depressed  I am moderately anxious or depressed  I am severely anxious or depressed  I am extremely anxious or depressed | 41 (40%)  28 (27%)  23 (22%)  8 (8%)  3 (3%) |
| **Visual analogue scale (VAS)** | **Mean (SD)** |
| Mean health status today | 69.6 (22.6) |
| **Visual analogue scale (VAS)** | **Median (IQR)** |
| Median health status today | 75 (50, 90) |
