# Supplementary material for: Evaluation of the psychometric properties of the Episodic Disability Questionnaire (EDQ) among women living with HIV in the United Kingdom: A self-reported repeated measure study
Source: PLoS One. 2026 May 4;21(5):e0336679. doi: 10.1371/journal.pone.0336679 (PMC13138649; doi:10.1371/journal.pone.0336679)
Supplement: S3 Table — (DOCX) [file pone.0336679.s003.docx]

**S3 Table. WHODAS 2.0 12-item frequency (%) of participants scoring per difficulty level**

| **WHODAS Item (*domain*)** | **None**  **n (%)** | **Mild**  **n (%)** | **Moderate**  **n (%)** | **Severe**  **n (%)** | **Extreme**  **n (%)** |
| --- | --- | --- | --- | --- | --- |
| In the last 30 days, how much difficulty did you have in: - **Standing for long periods** such as 30 minutes? (*mobility challenges*) | 40 (22.73) | 16 (9.09) | 25 (14.20) | 15 (8.52) | 8 (4.55) |
| In the last 30 days, how much difficulty did you have in: - Taking care of your **household responsibilities**? (*challenges to life activities*) | 40 (22.73) | 20 (11.36) | 30 (17.05) | 6 (3.41) | 8 (4.55) |
| In the last 30 days, how much difficulty did you have in: - **Learning a new** **task**, for example, learning how to get to a new place? (*cognitive health challenges*) | 52 (29.55) | 24 (13.64) | 15 (8.52) | 9 (5.11) | 4 (2.27) |
| In the last 30 days, how much difficulty did you have: - joining in **community activities** (for example, festivities, religious or other activities) in the same way as anyone else can? (*challenges to social participation*) | 53 (30.11) | 17 (9.66) | 17 (9.66) | 8 (4.55) | 9 (5.11) |
| In the last 30 days, how much have you been **emotionally affected by your health problems?** (*challenges to social participation*) | 26 (14.77) | 25 (14.20) | 27 (15.34) | 20 (11.36) | 6 (3.41) |
| In the last 30 days, how much difficulty did you have in: - **Concentrating on doing something for ten minutes**? (*cognitive health challenges*) | 53 (30.11) | 16 (9.09) | 24 (13.64) | 7 (3.98) | 4 (2.27) |
| In the last 30 days, how much difficulty did you have in: - **Walking a long distance** such as a kilometre [or equivalent]? (*mobility challenges*) | 46 (26.14) | 15 (8.52) | 20 (11.36) | 8 (4.55) | 15 (8.52) |
| In the last 30 days, how much difficulty did you have in: - **Washing your whole body**? (*challenges with self-care*) | 66 (37.50) | 13 (7.39) | 15 (8.52) | 9 (5.11) | 1 (0.57) |
| In the last 30 days, how much difficulty did you have in: - **Getting dressed**? (*challenges with self-care*) | 70 (39.77) | 11 (6.25) | 16 (9.09) | 6 (3.41) | 1 (0.57) |
| In the last 30 days, how much difficulty did you have in: - **Dealing with people you do not know**? (*challenges to getting along*) | 56 (31.82) | 17 (9.66) | 16 (9.09) | 12 (6.82) | 3 (1.70) |
| In the last 30 days, how much difficulty did you have in: - **Maintaining a friendship**? (*challenges to getting along*) | 62 (35.23) | 17 (9.66) | 16 (9.09) | 7 (3.98) | 2 (1.14) |
| In the last 30 days, how much difficulty did you have in: - Your **day-to-day work**? (*challenges to life activities*) | 52 (29.55) | 12 (6.82) | 26 (14.77) | 7 (3.98) | 7 (3.98) |
