# Supplementary material for: Evaluation of the psychometric properties of the Episodic Disability Questionnaire (EDQ) among women living with HIV in the United Kingdom: A self-reported repeated measure study
Source: PLoS One. 2026 May 4;21(5):e0336679. doi: 10.1371/journal.pone.0336679 (PMC13138649; doi:10.1371/journal.pone.0336679)
Supplement: S1 Fig — (PDF) [file pone.0336679.s004.pdf]

S1 Figure. Construct validity hypotheses testing and results of the Episodic Disability Questionnaire (EDQ)

Total number of hypotheses: 59 (6 primary, 53 secondary)

Total hypotheses met: 47/59 (80%)

Primary hypotheses (n=6)

World Health Organization Disability Assessment Schedule (WHODAS) sum score will moderately correlate (>0.50) with EDQ severity domains scores (hypotheses 1 to 6)

Domain	Spearman Correlation Coefficient	Lower Bound 95% Confidence Interval (CI)	Upper Bound 95% CI	Hypothesis Confirmed
**Physical**	0.79	0.69	0.86	Yes
**Cognitive**	0.74	0.62	0.82	Yes
**Mental-emotional**	0.77	0.66	0.84	Yes
**Uncertainty**	0.69	0.56	0.79	Yes
**Daily activities**	0.88	0.82	0.92	Yes
**Social inclusion**	0.80	0.70	0.87	Yes

Secondary hypotheses (n=53)

Work and Social Adjustment Scale (WSAS) sum score will strongly correlate (>0.70) with EDQ severity domain score (hypothesis 7)

Domain	Spearman Correlation Coefficient	Lower Bound 95% CI	Upper Bound 95% CI	Hypothesis Confirmed
**Mental-emotional**	0.72	0.49	0.81	No

WSAS sum score will moderately correlate (>0.50) with EDQ severity domain scores (hypotheses 8 to 9)

Domain	Spearman Correlation Coefficient	Lower Bound 95% CI	Upper Bound 95% CI	Hypothesis Confirmed
**Daily activities**	0.80	0.70	0.87	Yes
**Social inclusion**	0.80	0.70	0.87	Yes

WSAS sum score will strongly correlate (>0.70) with EDQ presence domain scores (hypotheses 10 to 12)

Domain	Spearman Correlation Coefficient	Lower Bound 95% CI	Upper Bound 95% CI	Hypothesis Confirmed
**Mental-emotional**	0.69	0.56	0.79	No
**Social inclusion**	0.76	0.65	0.84	No

WSAS sum score will moderately correlate (>0.50) with EDQ presence domain score

Domain	Spearman Correlation Coefficient	Lower Bound 95% CI	Upper Bound 95% CI	Hypothesis Confirmed
**Daily activities**	0.81	0.71	0.87	Yes

EQ-5D-5L mobility score will strongly correlate (>0.70) with EDQ severity domain score (hypothesis 13)

Domain	Spearman Correlation	Lower Bound 95% CI	Upper Bound 95% CI	Hypothesis Confirmed
**Physical**	0.55	0.39	0.68	No

EQ-5D-5L mobility score will strongly correlate (>0.70) with EDQ presence domain score (hypothesis 14)

Domain	Spearman Correlation	Lower Bound 95% CI	Upper Bound 95% CI	Hypothesis Confirmed
**Physical**	0.46	0.28	0.61	No

EQ-5D-5L self-care score will strongly correlate (>0.70) with EDQ severity domain score (hypothesis 15)

Domain	Spearman Correlation	Lower Bound 95% CI	Upper Bound 95% CI	Hypothesis Confirmed
**Daily activities**	0.70	0.57	0.79	No

EQ-5D-5L self-care score will moderately correlate (>0.50) with EDQ presence domain score (hypothesis 16)

Domain	Spearman Correlation	Lower Bound 95% CI	Upper Bound 95% CI	Hypothesis Confirmed
**Daily activities**	0.69	0.56	0.79	Yes

EQ-5D-5L usual activity score will moderately correlate (>0.50) with EDQ severity domain score (hypothesis 17)

Domain	Spearman Correlation	Lower Bound 95% CI	Upper Bound 95% CI	Hypothesis Confirmed
**Social inclusion**	0.71	0.58	0.80	Yes

EQ-5D-5L usual activity score will moderately correlate (>0.50) with EDQ presence domain score (hypothesis 18)

Domain	Spearman Correlation	Lower Bound 95% CI	Upper Bound 95% CI	Hypothesis Confirmed
**Social inclusion**	0.63	0.49	0.74	No

EQ-5D-5L pain score will moderately correlate (>0.50) with EDQ severity domain score (hypothesis 19)

Domain	Spearman Correlation	Lower Bound 95% CI	Upper Bound 95% CI	Hypothesis Confirmed
**Physical**	0.76	0.65	0.84	Yes

EQ-5D-5L pain score will strongly correlate (>0.70) with EDQ presence domain score (hypothesis 20)

Domain	Spearman Correlation	Lower Bound 95% CI	Upper Bound 95% CI	Hypothesis Confirmed
**Physical**	0.63	0.48	0.74	No

EQ-5D-5L anxiety/depression score will strongly correlate (>0.70) with EDQ severity domain score (hypothesis 21)

Domain	Spearman Correlation	Lower Bound 95% CI	Upper Bound 95% CI	Hypothesis Confirmed
**Mental-emotional**	0.75	0.64	0.83	No

Participants meeting the Equality Act Disability Definition (EADD) will have significantly higher EDQ severity domain score compared to those not meeting the EADD disability definition (hypothesis 22)

Domain	Wilcox Test	*p* value	Hypothesis Confirmed
**Daily activities**	291.5	<0.001	Yes

Participants meeting the EADD will have significantly higher EDQ presence domain score compared to those not meeting the EADD disability definition (hypothesis 23)

Domain	Wilcox Test	*p* value	Hypothesis Confirmed
**Daily activities**	330.5	<0.001	Yes

Participants who completed T1 EDQ on a ‘good day’ will have significantly lower EDQ severity domain scores compared to those who completed T1 EDQ on a ‘bad day’ (hypotheses 24 to 29)

**Domain**	**Wilcox Test**	***p* value**	**Hypothesis Confirmed**
**Physical**	387	<0.001	Yes
**Cognitive**	427	<0.001	Yes
**Mental-emotional**	376.5	<0.001	Yes
**Uncertainty**	347.5	<0.001	Yes
**Daily activities**	293.5	<0.001	Yes
**Social inclusion**	439.5	<0.001	Yes

Participants who completed T1 EDQ on a ‘good day’ will have significantly lower EDQ presence domain scores compared to those who completed T1 EDQ on a ‘bad day’ (hypotheses 30 to 35)

**Domain**	**Wilcox Test**	***p* value**	**Hypothesis Confirmed**
**Physical**	486.5	0.003	Yes
**Cognitive**	486	0.002	Yes
**Mental-emotional**	433.5	<0.001	Yes
**Uncertainty**	479	0.002	Yes
**Daily activities**	294.5	<0.001	Yes
**Social inclusion**	489.5	0.004	Yes

Participants with two or more concurrent health conditions (multimorbidity) will have significantly higher EDQ severity domain scores (hypotheses 36 to 41)

**Domain**	**Wilcox Test**	***p* value**	**Hypothesis Confirmed**
**Physical**	338.5	0.002	Yes
**Cognitive**	437	0.03	Yes
**Mental-emotional**	397	0.01	Yes
**Uncertainty**	432	0.03	Yes
**Daily activities**	370.5	0.005	Yes
**Social inclusion**	422.5	0.02	Yes

Participants with two or more concurrent health conditions (multimorbidity) will have significantly higher EDQ presence domain scores (hypotheses 42 to 47)

**Domain**	**Wilcox Test**	***p* value**	**Hypothesis Confirmed**
**Physical**	378	0.007	Yes
**Cognitive**	426.5	0.02	Yes
**Mental-emotional**	390	0.008	Yes
**Uncertainty**	546.5	0.24	No
**Daily activities**	407.5	0.01	Yes
**Social inclusion**	441.5	0.04	Yes

Economically inactive participants will have significantly higher EDQ severity domain scores than economically active participants (hypotheses 48 to 53)

**Domain**	**Wilcox Test**	***p* value**	**Hypothesis Confirmed**
**Physical**	605	<0.001	Yes
**Cognitive**	553	<0.001	Yes
**Mental-emotional**	622	0.001	Yes
**Uncertainty**	804	0.06	No
**Daily activities**	614	<0.001	Yes
**Social inclusion**	666	0.003	Yes

Participants receiving benefits (social security) will have significantly higher EDQ severity domain scores than participants who do not receive benefits (hypotheses 54 to 59)

**Domain**	**Wilcox Test**	***p* value**	**Hypothesis Confirmed**
**Physical**	574	<0.001	Yes
**Cognitive**	439	<0.001	Yes
**Mental-emotional**	609	<0.001	Yes
**Uncertainty**	545.5	<0.001	Yes
**Daily activities**	565.5	<0.001	Yes
**Social inclusion**	574	<0.001	Yes
