# Supplementary material for: Evaluation of the psychometric properties of the Episodic Disability Questionnaire (EDQ) among women living with HIV in the United Kingdom: A self-reported repeated measure study
Source: PLoS One. 2026 May 4;21(5):e0336679. doi: 10.1371/journal.pone.0336679 (PMC13138649; doi:10.1371/journal.pone.0336679)
Supplement: Attachment — Submitted filename: Responce to Reviewers.docx [file pone.0336679.s006.docx]

# RESPONSE TO REVIEWERS

PONE-D-25-57930

Evaluation of the psychometric properties of the Episodic Disability Questionnaire (EDQ) among women living with HIV in the United Kingdom: a self-reported repeated measure study

We would like to sincerely thank the reviewers for their thoughtful, constructive, and engaged feedback on our manuscript. We greatly appreciate the time and expertise invested in reviewing our work. The comments were insightful and have strengthened the clarity, rigour, and overall contribution of the paper.

We have amended the manuscript to include the HIV Action Plan 2025-2030 (citation 21, lines 94-96, 99-100 and 492-493) published in December 2025, following our original submission. We have included two new citations from the UK Government’s Disability Unit (citation 20, lines 92-94; and citation 66, line 493). We have also included an additional citation to strengthen the discussion chapter (citation 58, lines 429-431 and 449-452). We have included an in-depth discussion on the magnitude of disability relative to population norms (lines 448-457). We have reordered the discussion section to match the flow of the results: internal consistency, test-retest reliability, measurement precision, construct validity, disability prevalence, and disability profile. We have also amended the conclusion to include outcomes for prevalence and severity in the discussion (lines 524-525), reflected in the abstract (lines 61-62).

We enclose, along with this response to reviewer's letter, the following items:

- Manuscript
- Revised Manuscript with Tracked Changes
- S1 Table
- S2 Table
- S3 Table
- S1 Figure

We have carefully considered each point raised and have revised the manuscript accordingly. Below, we provide a detailed, point-by-point response outlining how each comment has been addressed.

## Journal Requirements

**Review 1:** Please ensure that your manuscript meets PLOS ONE's style requirements, including those for file naming. The PLOS ONE style templates can be found at

<https://journals.plos.org/plosone/s/file?id=wjVg/PLOSOne_formatting_sample_main_body.pdf> and

[https://journals.plos.org/plosone/s/file?id=ba62/PLOSOne_formatting_sample_title_authors_affiliations.pdf](https://gbr01.safelinks.protection.outlook.com/?url=https%3A%2F%2Ftrack.editorialmanager.com%2FCL0%2Fhttps%3A%252F%252Fjournals.plos.org%252Fplosone%252Fs%252Ffile%253Fid%3Dba62%252FPLOSOne_formatting_sample_title_authors_affiliations.pdf%2F1%2F010f019bda1b5e83-b4358d4f-8074-447a-befc-d982fbe62897-000000%2FO5cIZzDQPdhQ-IBnM_LanxEnT1Lq1Y6kMK9QLbGD8Ww%3D246&data=05%7C02%7Cdarren.brown11%40nhs.net%7C765ee5c6b8dc4b9b1a9308de57edc5ab%7C37c354b285b047f5b22207b48d774ee3%7C0%7C1%7C639044875904666527%7CUnknown%7CTWFpbGZsb3d8eyJFbXB0eU1hcGkiOnRydWUsIlYiOiIwLjAuMDAwMCIsIlAiOiJXaW4zMiIsIkFOIjoiTWFpbCIsIldUIjoyfQ%3D%3D%7C0%7C%7C%7C&sdata=MSJC6dBV1UX5y4EFWSx5B39McKSI01yaoHvE0x1KHAA%3D&reserved=0)

**Response 1: Thank you. The manuscript and supporting information have been amended to meet PLOS ONE’s style requirements for formatting, authorship, tables, figures, and file naming.**

**Review 2:** We noticed you have some minor occurrence of overlapping text with the following previous publication(s), which needs to be addressed:

- <https://doi.org/10.1186/s12879-023-08958-7>

In your revision ensure you cite all your sources (including your own works), and quote or rephrase any duplicated text outside the methods section. Further consideration is dependent on these concerns being addressed.

**Response 2: Thank you. We have addressed occurrences of any overlapping text and rephrased any duplications throughout the manuscript, ensuring to cite work, including our own, appropriately. This is demonstrated via tracked changes in the revised manuscript.**

**Review 3:** Please note that funding information should not appear in any section or other areas of your manuscript. We will only publish funding information present in the Funding Statement section of the online submission form. Please remove any funding-related text from the manuscript.

**Response 3: Thank you. Funding information has been removed from the title page.**

**Review 4:** We note that the grant information you provided in the ‘Funding Information’ and ‘Financial Disclosure’ sections do not match.

When you resubmit, please ensure that you provide the correct grant numbers for the awards you received for your study in the ‘Funding Information’ section.

**Response 4: Thank you for identifying this error. We confirm that the study was funded by a British HIV Association (BHIVA) Research Award with grant number: BHIVA/5020/2020/Brown.** **We have also addressed this** **in the ‘Funding Information’ section.**

**Review 5:** We note that your Data Availability Statement is currently as follows: All relevant data are within the manuscript and in Supporting Information files.

Please confirm at this time whether or not your submission contains all raw data required to replicate the results of your study. Authors must share the “minimal data set” for their submission. PLOS defines the minimal data set to consist of the data required to replicate all study findings reported in the article, as well as related metadata and methods (<https://journals.plos.org/plosone/s/data-availability#loc-minimal-data-set-definition>).

For example, authors should submit the following data:

- The values behind the means, standard deviations and other measures reported;

- The values used to build graphs;

- The points extracted from images for analysis.

Authors do not need to submit their entire data set if only a portion of the data was used in the reported study.

If your submission does not contain these data, please either upload them as Supporting Information files or deposit them to a stable, public repository and provide us with the relevant URLs, DOIs, or accession numbers. For a list of recommended repositories, please see <https://journals.plos.org/plosone/s/recommended-repositories>.

If there are ethical or legal restrictions on sharing a de-identified data set, please explain them in detail (e.g., data contain potentially sensitive information, data are owned by a third-party organization, etc.) and who has imposed them (e.g., an ethics committee). Please also provide contact information for a data access committee, ethics committee, or other institutional body to which data requests may be sent. If data are owned by a third party, please indicate how others may request data access.

**Response 5: Thank you. Anonymised raw data required to replicate the psychometric analyses have been submitted to a public data repository, pending publication and accessible via the following URL: <https://doi.org/10.5061/dryad.tht76hfdd>**

**Review 6:** Please include captions for your Supporting Information files at the end of your manuscript, and update any in-text citations to match accordingly. Please see our Supporting Information guidelines for more information: <http://journals.plos.org/plosone/s/supporting-information>.

**Response 6: Thank you. Supporting information has been included at the end of the manuscript and in-text citations match accordingly.**

**Review 7:** If the reviewer comments include a recommendation to cite specific previously published works, please review and evaluate these publications to determine whether they are relevant and should be cited. There is no requirement to cite these works unless the editor has indicated otherwise.

**Response 7: Thank you. Reviewers’ recommendations have been taken into careful consideration. We did not conduct a Rasch analysis, therefore we have not included Reviewer #2 suggested references which related to Rasch analysis.**

## Reviewer #1

**Review 8:** Overall Assessment: The manuscript addresses a critical area in HIV care and women's health. The specific focus on women living with HIV, and the use of the EDQ to capture episodic disability, makes it distinctive and impactful. The adherence to COSMIN guidelines and the comprehensive psychometric evaluation are definite strengths.

**Response 8: Thank you for your review and feedback.**

**Review 9:** High Clinical and Social Relevance: The study addresses disability in women living with HIV, a population often underrepresented in research, particularly concerning episodic disability and multimorbidity. The focus on aging with HIV further enhances its relevance.

**Response 9: Thank you for your review and feedback.**

**Review 10:** Strong Methodological Rigor:
o COSMIN Adherence: Explicitly stating adherence to COSMIN guidelines indicates a high standard for psychometric evaluation.
o Repeated Measures Design: The two-timepoint data collection for test-retest reliability is crucial for stability assessment.
o Use of Criterion Measures: Employing well-established measures like WHODAS 2.0, EQ-5D-5L, and WSAS for construct validity strengthens the findings.

**Response 10: Thank you for your review and feedback.**

**Review 11:** Specific Population Focus: Targeting women living with HIV in the UK allows for nuanced insights into their specific experiences of disability, contrasting with previous studies predominantly on men.

**Response 11: Thank you for your review and feedback.**

**Review 12:** Abstract Conciseness and Impact:
- Issue: The abstract, particularly the Methods and Results sections, is quite dense and could be streamlined to enhance clarity and impact for high-impact journals where abstracts are often read first. Phrases like "Cronbach alpha ≥0.7" or "ICC >0.7" are vague when specific ranges are available.
- Suggestion:
- Methods: Instead of "Cronbach alpha ≥0.7," state the observed range (e.g., "Cronbach alpha ranged from 0.83 to 0.92"). Similarly, use the observed ICC range.
- Results: Quantify "varied precision" (e.g., "Precision varied, highest in daily activities (MDC95%: 6.10) and lowest in mental-emotional (MDC95%: 11.52)").
- Typos: Correct the obvious typo "Cronbach alpha 30.7" to "≥0.7" (or the actual range) in the abstract.
- Overall: Aim for more active voice and direct phrasing to convey key findings efficiently.

**Response 12: Thank you for your review and constructive feedback. We have removed from the abstract methods the acceptable threshold for internal consistency (Cronbach’s alpha ≥0.7), acceptable threshold for lower bound confidence intervals for test-retest reliability (ICC > 0.7), and threshold for construct validity ( ≥75% a priori hypothesis met) (lines 47-49). These were the thresholds for psychometric evaluation. We have reserved reporting the observed range within results (lines 54-58). We have updated precision results to act on your recommendations (lines 56-57). The results do identify “Cronbach’s alpha ranged from 0.83 (social domain) to 0.92 (daily activities domain)” (lines 54-55). We have modified the text to aim for more active voice and direct phrasing throughout the abstract.**

**Review 13:** Clarity on Construct Validity (Methods Section):
- Issue: The methods section states, "We examined correlations for 59 total a priori hypotheses: 6 primary and 53 secondary." While the outcome ("80%... hypotheses met") is in the abstract and results, the process of how these hypotheses were generated and tested is not fully detailed in the Methods.
- Suggestion: Briefly describe the nature of these a priori hypotheses in the Methods section. For example, "A priori hypotheses were formulated based on previous EDQ validation studies and clinical understanding, predicting specific correlational patterns between EDQ domains and the criterion measures (WHODAS 2.0, EQ-5D-5L, WSAS), as well as known group differences (e.g., by concurrent health conditions)." Referencing the Supplementary File for the full list is good, but a summary of how they were tested (e.g., using Spearman correlations and Wilcoxon rank sum tests, as later mentioned implicitly) should be here.

**Response 13: Thank you for your review and constructive feedback. We have modified the methods for construct validity (lines 243-260) to include suggestions for the formulation of a prior hypothesis (lines 244-246) and methods to calculate Spearman correlation coefficients to test associations between continuous EDQ scores and criterion measures (lines 252-254), and Wilcoxon rank sum tests to compare EDQ scores across ordinal criterion measures (lines 254-255).** **We have also identified that the full list of *a priori* hypotheses is available in S1 Figure (line 252).**

**Review 14:** Measurement Precision Interpretation:
- Issue: The section on MDC95% provides the definition and values but could benefit from a clearer interpretation of what these values mean for the EDQ in practical terms. What does an MDC95% of 6.10 versus 11.52 imply for detecting meaningful change in clinical practice or research?
- Suggestion: In the "Measurement Precision" section of the Methods, or in the Discussion when elaborating on precision results, add a sentence or two explaining the practical implications of these MDC values. For example, "A lower MDC95% indicates greater precision, meaning smaller changes in scores can be confidently considered true changes rather than measurement error. Thus, the EDQ is most precise in detecting changes in [domain with lowest MDC] and least precise in [domain with highest MDC]."

**Response 14: Thank you for your review and constructive feedback. We have modified the discussion to include suggested recommendations for interpreting precision and MDC95% scores (lines 410-413).**

**Review 15:** Discussion of Episodic Scale Test-Retest:
- Issue: The methods state, "Our test-retest assessment focused on EDQ presence and severity scales, as the episodic scale refers to fluctuations in disability in the past week, and we did not expect consistency in this scale." However, Table 3 does present ICCs for the EDQ Episodic scale, some of which are quite low (e.g., Daily activities: 0.20).
- Suggestion: Reconcile this. Either remove the episodic scale ICCs from Table 3 if consistency was genuinely not expected and not relevant, or, more likely, explicitly discuss these lower ICCs in the Results and Discussion. If they are low because the scale is designed to capture fluctuations, then explain that this reflects the nature of episodic disability and is not necessarily a "failure" of reliability for that specific scale component, but rather an expected outcome demonstrating its sensitivity to change. This would strengthen the understanding of episodic disability measurement.

**Response 15: Thank you for your review and constructive feedback. We have modified the discussion to include suggested recommendations for reporting and interpreting lower ICCs within the episodic scale (lines 395-400). We have consequently retained the EDQ episodic domains ICC (95% CI) results within Table 3 (lines 331-334).**

**Review 16:** Deeper Discussion of Limitations and Future Research:
- Issue: While limitations are acknowledged, the discussion could expand on how these limitations affect interpretation and what specific future research is needed. For example, the limitation about electronic administration not being generalizable to paper-based could be explored further.
- Suggestion: For each key limitation (e.g., self-report, electronic administration, sample size for broader populations), suggest concrete future studies or methodological approaches. For instance: "Future research should explore the EDQ's performance when administered via paper-and-pencil, as accessibility for all populations may require mixed-mode administration, and investigate potential mode effects." Or "Longitudinal studies using the EDQ are necessary to further confirm its responsiveness to change over longer periods and to establish minimal clinically important difference (MCID) values."

**Response 16: Thank you for your review and constructive feedback. We have modified the discussion strengths and limitations section (lines 501-520) to include suggested recommendations, providing interpretation and future recommendations for research.**

**Review 17:** Minor Presentation Points:
- "Table 1: Participants' demographic, health, and social characteristics": This table is very extensive. While good for transparency, consider if some less critical categories can be moved to supplementary material to keep the main text focused, especially if space is a concern for a high-impact journal.

**Response 17: Thank you for your review and constructive feedback. We have modified Table 1 (lines 300-302) to streamline the included results and located additional characteristics in a new supporting information S1 Table.**

**Review 18:** By addressing these points, the manuscript will be greatly enhanced its readability for publication.

**Response 18: Thank you for your review and constructive feedback, which have greatly enhanced the manuscript’s readability and interpretation.**

## Reviewer #2

**Review 19:** The article entitled “Evaluation of the psychometric properties of the Episodic Disability Questionnaire (EDQ) among women living with HIV in the United Kingdom: a self-reported repeated measure study” evaluates the psychometric properties of the EDQ among women living with HIV in the United Kingdom using both classical analyses and analyses within the framework of Rasch models. On the whole, the article is clear and well-written, and it fulfils the aims and scopes of PLOS ONE. However, in my opinion some, modifications are needed for the article to be published.

**Response 19: Thank you for your review and constructive feedback. This study aimed to assess the EDQ for its ability to measure disability experienced by women living with HIV in the UK, specifically internal consistency, test-retest reliability, precision of measurement, and construct validity. Rasch analysis was not included within this research; however was conducted at an earlier stage in the development of the EDQ:** [**https://link.springer.com/article/10.1186/s12955-020-01643-2**](https://link.springer.com/article/10.1186/s12955-020-01643-2)

**We have modified the manuscript to ensure the text states “*Severity and presence domain scores are calculated using the algorithm derived from the previous Rasch analysis*” (lines152-153) and “*The EDQ was derived from the preceding SF-HDQ*” (line 151) and** **“*Severity and presence domain scores (range: 0 to 100) were calculated using the algorithm established from the previous Rasch analysis*” (lines 213-214).**

**Review 20:** The main point that deserves attention is the part concerning the Rasch analyses. By reading the section “Results”, it seems to me that traditional analyses were run on each of the six domains of the EDQ, separately for severity, presence, and episodic nature of disability. Rasch analyses were also run on each of the six dimensions, separately for severity and presence, whereas Rasch analyses were not run for episodic nature of disability. This understanding, which I do not know if it is correct or not, resulted from reading the section “Results”, but not from the previous sections “Questionnaires” and “Analysis”. Moreover, several aspects concerning the Rasch analyses are missing and/or should be better clarified:

To which Rasch analysis algorithms do the authors refer on line 195? I think the authors used the Rasch model for dichotomous items for the analysis of presence and episodic score, and a Rasch model for polytomous items for the analysis of severity but, with respect to the latter, it is not clear if they used the rating scale model (Andrich, 1978) or the partial credit model (Masters, 1982). The authors should make this clear.

**Response 20: Thank you for your review and query. This study aimed to assess the EDQ for its ability to measure disability experienced by women living with HIV in the UK, specifically internal consistency, test-retest reliability, precision of measurement, and construct validity. Rasch analysis was not included within this research; however was conducted at an earlier stage in the development of the EDQ:** [**https://link.springer.com/article/10.1186/s12955-020-01643-2**](https://link.springer.com/article/10.1186/s12955-020-01643-2)

**We have modified the manuscript to ensure the text states “*Severity and presence domain scores are calculated using the algorithm derived from the previous Rasch analysis*” (lines152-153) and “*The EDQ was derived from the preceding SF-HDQ*” (line 151) and** **“*Severity and presence domain scores (range: 0 to 100) were calculated using the algorithm established from the previous Rasch analysis*” (lines 213-214).**

**Review 21:** The authors should explain to which scores they refer when writing “score range: 0-100” in lines 195-196. From this paragraph, my guess is that the authors refer to linear transformations of the ability/health measures of individuals obtained by applying the Rasch model but, from line 257, it seems that the raw score are transformed to range from 0 to 100.

**Response 21: Thank you for your review, query, and constructive feedback. Rasch analysis was not included within this research.**

**We have modified the manuscript to ensure the text states “*Severity and presence domain scores are calculated using the algorithm derived from the previous Rasch analysis*” (lines152-153) and “*The EDQ was derived from the preceding SF-HDQ*” (line 151) and** **“*Severity and presence domain scores (range: 0 to 100) were calculated using the algorithm established from the previous Rasch analysis*” (lines 213-214). citing the previous work:** [**https://link.springer.com/article/10.1186/s12955-020-01643-2**](https://link.springer.com/article/10.1186/s12955-020-01643-2)

**We have modified the manuscript to state “*Episodic domain scores (range: 0 to 100) included a simple sum transformation*” (lines 214-215) citing previous work describing this process:**

[**https://link.springer.com/article/10.1186/s12879-023-08958-7**](https://link.springer.com/article/10.1186/s12879-023-08958-7)

**Review 22:** Were the assumptions of the Rasch models verified? Specifically, did the authors verify that the six dimensions of the EDQ, in both the analysis on severity and the analysis of presence, were unidimensional? If they did not, the authors could use principal component analysis of the residuals to investigate unidimensionality (Smith, 2002). Did the authors verify the assumption of local independence?

**Response 22: Thank you for your review, query, and constructive feedback. Rasch analysis was not included within this research.**

**We have modified the manuscript to ensure the text states “*Severity and presence domain scores are calculated using the algorithm derived from the previous Rasch analysis*” (lines152-153) and “*The EDQ was derived from the preceding SF-HDQ*” (line 151) and** **“*Severity and presence domain scores (range: 0 to 100) were calculated using the algorithm established from the previous Rasch analysis*” (lines 213-214), citing the previous work:** [**https://link.springer.com/article/10.1186/s12955-020-01643-2**](https://link.springer.com/article/10.1186/s12955-020-01643-2)

**Review 23:** A Rasch analysis of polytomous items, conducted either with the rating scale model or with the partial credit model, allows for investigating if the rating scale functions as intended, that is, if higher response categories actually reflect higher levels of the latent variable. To this aim, it is needed that the thresholds (i.e., the points on the latent variable at which two adjacent categories are equally likely) are ordered increasingly (Andrich, 2010, 2011; Colledani et al., 2025). The authors should state if, in the Rasch analysis on polytomous items, the thresholds were ordered increasingly.

**Response 23: Thank you for your review, query, and constructive feedback. Rasch analysis was not included within this research.**

**We have modified the manuscript to ensure the text states “*Severity and presence domain scores are calculated using the algorithm derived from the previous Rasch analysis*” (lines152-153) and “*The EDQ was derived from the preceding SF-HDQ*” (line 151) and** **“*Severity and presence domain scores (range: 0 to 100) were calculated using the algorithm established from the previous Rasch analysis*” (lines 213-214), citing the previous work:** [**https://link.springer.com/article/10.1186/s12955-020-01643-2**](https://link.springer.com/article/10.1186/s12955-020-01643-2)

**Review 24:** The authors should add and interpret Rasch-based statistics of the functioning of the items such as outfit and/or infit (Linacre, 2002).

**Response 24: Thank you for your review, query, and constructive feedback. Rasch analysis was not included within this research, therefore we cannot interpret Rasch statistics of the functioning of items such as outfit and/or infit.**

**We have modified the manuscript to ensure the text states “*Severity and presence domain scores are calculated using the algorithm derived from the previous Rasch analysis*” (lines152-153) and “*The EDQ was derived from the preceding SF-HDQ*” (line 151) and** **“*Severity and presence domain scores (range: 0 to 100) were calculated using the algorithm established from the previous Rasch analysis*” (lines 213-214), citing the previous work:** [**https://link.springer.com/article/10.1186/s12955-020-01643-2**](https://link.springer.com/article/10.1186/s12955-020-01643-2)

**Review 25:** The authors investigated internal consistency using Cronbach’s α (in the analysis of polytomous items) and KR-20 (in the analysis of dichotomous items). Since the authors also estimated Rasch models, they could report the Rasch-based measure of internal consistency called person-separation reliability, which applies to both dichotomous and polytomous items and has been found to be a better estimate of internal consistency than Cronbach’s α and KR-20 (Anselmi et al., 2019).

**Response 25: Thank you for your review, query, and constructive feedback. We calculated Cronbach’s alpha (severity domain) and Kuder-Richardson-20 (KR-20) (presence and episodic domains) for time 1 (T1) with 95% confidence intervals (CI) (≥0.7 acceptable) (lines 221-223).**

**We did not estimate Rasch models within this research, therefore cannot perform person-separation reliability instead of Cronbach’s alpha and KR-20.**

**We have modified the manuscript to ensure the text states “*Severity and presence domain scores are calculated using the algorithm derived from the previous Rasch analysis*” (lines152-153) and “*The EDQ was derived from the preceding SF-HDQ*” (line 151) and** **“*Severity and presence domain scores (range: 0 to 100) were calculated using the algorithm established from the previous Rasch analysis*” (lines 213-214), citing the previous work:** [**https://link.springer.com/article/10.1186/s12955-020-01643-2**](https://link.springer.com/article/10.1186/s12955-020-01643-2)

**Review 26:** References
Andrich, D. (1978). A rating scale formulation for ordered response categories. Psychometrika, 43(4), 561–573. <https://doi.org/10.1007/BF02293814>

Andrich, D. (2010). Understanding the response structure and process in the polytomous Rasch model. In M. L. Nering & R. Ostini (Eds.), Handbook of polytomous item response theory models (pp. 123-152). Routledge. <https://doi.org/10.4324/9780203861264>

Andrich, D. (2011). Rating scales and Rasch measurement. Expert Review of Pharmacoeconomics & Outcomes Research, 11(5), 571–585. <https://doi.org/10.1586/erp.11.59>

Anselmi, P., Colledani, D., & Robusto, E. (2019). A Comparison of classical and modern measures of internal consistency. Frontiers in Psychology, 10, 2714. <https://doi.org/10.3389/fpsyg.2019.02714>

Colledani, D., González Pizzio, A. P., Devita, M., & Anselmi, P. (2025). Investigating the Functioning of Rating Scales with Rasch Models. Assessment, 32(3), 434–446. <https://doi.org/10.1177/10731911241245792>

Linacre, J. M. (2002). What do Infit and Outfit, Mean-square and Standardized mean? Rasch Measurement Transactions, 16(2), 878.

Masters, G. N. (1982). A Rasch model for partial credit scoring. Psychometrika, 47,149–174. <http://dx.doi.org/10.1007/BF02296272>

Smith, E. V., Jr., (2002). Detecting and evaluating the impact of multidimensionality using item fit statistics and principal component analysis of residuals. Journal of Applied Measurement, 3(2), 205–231.

**Response 26: Thank you for your review and suggested references. Rasch analysis was not included within this research, however was conducted at an earlier stage in the development of the EDQ:** [**https://link.springer.com/article/10.1186/s12955-020-01643-2**](https://link.springer.com/article/10.1186/s12955-020-01643-2)

**The recommended references relate to Rasch analysis, therefore have not been included into this manuscript.**

**Review 27:** Lines 287-288. It is written “We derived a priori hypotheses from earlier construct validity assessments of the EDQ [10].” The authors should briefly provide a description of their a priori hypotheses and how they were derived from earlier construct validity assessments of the EDQ

**Response 27: Thank you for your review and constructive feedback. We have modified the methods for construct validity (lines 243-260) to include suggestions for the formulation of a prior hypothesis (lines 244-246) and methods to calculate Spearman correlation coefficients to test associations between continuous EDQ scores and criterion measures (lines 252-254), and Wilcoxon rank sum tests to compare EDQ scores across ordinal criterion measures (lines 254-255).** **We have also identified that the full list of *a priori* hypotheses is available in S1 Figure (line 252).**

**Review 28:** Line 130: The authors could provide a brief definition of “episodic disability”.

**Response 28: Thank you for your review and constructive feedback. We have modified the introduction to include a definition of episodic disability: “*Episodic disability is conceptualised as any health-related challenges that may fluctuate or persist over time when living with a health condition*” (lines 83-84), citing the following references:**

[**https://link.springer.com/article/10.1186/1477-7525-6-76**](https://link.springer.com/article/10.1186/1477-7525-6-76)

[**https://www.tandfonline.com/doi/full/10.3109/09638288.2013.793408**](https://www.tandfonline.com/doi/full/10.3109/09638288.2013.793408)

**Review 29:** Lines 152-155: The authors should provide a brief description of the guidelines they refer to.

**Response 29: Thank you for your review and constructive feedback. We have modified the methods to state: “*We followed established COnsensus‐based Standards for the selection of health Measurement Instruments (COSMIN) guidelines, a set of consensus-based reporting recommendations for primary studies of the measurement properties of patient-reported outcome measures, to evaluate and report the measurement properties of the EDQ*” (lines 108-112).**

**Review 30:** Lines 235-236: It is written “The WSAS is a unidimensional scale, suggesting the scores cannot be compared across groups of different health conditions”. To my understanding, it is stated that the unidimensional nature of the WSAS suggests that the scores cannot be compared across groups of different health conditions. Is my understanding correct? If so, the authors should clarify why the unidimensional nature of the WSAS suggests that the scores cannot be compared across groups of different health conditions.

**Response 30: Thank you for your review and constructive feedback. We have modified the methods to state: “*The WSAS has demonstrated high internal consistency among people living with HIV and is used to assess functional impairment among people living with HIV and painful peripheral neuropathy*” (lines 192-194).**

**The context of being a unidimensional scale of perceived impairment in work or social functioning resulting from a health problem, is addressed in lines 186-187.** **We did not aim to compare disability between different health conditions therefore this text has been removed.**

**Review 31:** Line 262. If the authors used R packages, they should make explicit which packages they used.

**Response 31: Thank you for your review and constructive feedback. We have modified the methods to state: “*Analyses were conducted in R version 4.4.0 using the psych package*” (lines 219-220).**

**Review 32:** Line 386: “were;” should be replaced with “were:”.

**Response 32: Thank you for your review and constructive feedback. This recommendation has been actioned (line 359).**

**Review 33:** In the Section “Strengths and Limitations”, the authors should make more explicit which of the discussed aspects are strengths and which are limitations.

**Response 33: Thank you for your review and constructive feedback. We have modified the discussion strengths and limitations section (lines 501-520) to include suggested recommendations, providing interpretation and future recommendations for research.**

We thank the reviewers once again for their thoughtful and constructive feedback. The revisions prompted by these comments have strengthened the clarity, rigour, and overall contribution of the manuscript. We hope the changes made address all concerns satisfactorily and we appreciate the opportunity to improve the work through this review process.

Darren A Brown OBE BSc MSc MRes FCSP

25-Feb-2026
